# Naphthol Blue Black and ^99m^Tc-Labeled Mannosylated Human Serum Albumin (^99m^Tc-MSA) Conjugate as a Multimodal Lymph Node Mapping Nanocarrier

**DOI:** 10.1038/s41598-018-31933-1

**Published:** 2018-09-11

**Authors:** Ji Youn Lee, Ho Young Kim, Yun-Sang Lee, Jae Min Jeong

**Affiliations:** 1 Department of Nuclear Medicine, Institute of Radiation Medicine, Seoul National University College of Medicine, Seoul, Republic of Korea; 2 Department of Biomedical Sciences, Seoul National University Graduate School, Seoul, Republic of Korea; 3 Cancer Research Institute, Seoul National University, Seoul, Republic of Korea

## Abstract

^99m^Tc-labeled mannosylated human serum albumin (MSA) has been reported as a sentinel lymph node (SLN)-imaging agent by binding to macrophages in the LNs. By conjugating it with blue dye, we developed a new multimodal radio-nanocarrier by visual investigation, fluorescence imaging, and single photon emission computed tomography (SPECT)/computed tomography (CT). Binding affinities of seven blue dyes to MSA were tested. According to the spectroscopic study and visual inspection of MSA-bound dyes, naphthol blue black (NBB) was selected as the best candidate of multimodal agent. Thus, ^99m^Tc-MSA-NBB conjugate was prepared and further investigated using mice. After footpad injection, it showed high popliteal LN accumulation at 1 h. SPECT/CT also showed high popliteal as well as inguinal LN uptakes at 10 min that sustained until 2 h. In conclusion, we prepared a multimodal SLN imaging radio-nanocarrier, ^99m^Tc-MSA-NBB conjugate, and confirmed its excellency as a multimodal probe for SLN mapping.

## Introduction

The sentinel lymph node (SLN) is the first regional lymph node (LN) receiving lymphatic flow from the primary tumor. The detection of SLN is important to estimate tumor staging and for therapeutic decision-making in breast cancer and melanoma patients^1–4^.

Blue dyes^1,2^ or radiotracers^5–7^ have been used for the detection of SLN; however, both of these methods have limitations. Although blue dyes may visualize SLN directly during surgery and allow accurate identification of SLN without any special instrument or device^1,2^, these dyes are invisible under the skin or other tissues. Moreover, blue dyes rapidly diffuse to LNs adjacent to SLN due to small size (<2 nm), thereby posing difficulty in detection. Radiotracers such as ^99m^Tc-sulfur colloid may effectively identify the localization of SLN^5–7^ but necessitates the requirement of experienced surgeons to increase SLN detection rate and accuracy^8,9^. In addition, the portion of radiocolloids entering the lymphatic system is very low and the majority remains in the interstitial tissue of the injected site due to large particle size (~100 nm). To increase the efficiency of SLN detection, mannose receptor imaging radiotracers with small size (<10 nm) such as ^99m^Tc-mannosylated human serum albumin (MSA)^10,11^ and ^99m^Tc-mannosylated dextran (Lymphoseek) have been developed^12–14^.

The combination method of a radiotracer and blue dye method^4,15–18^ was used for SLN mapping to exploit the advantages associated with the two methods^15,19^. In breast cancer patients, the detection rate increased from 73% to 92%, and false negative ratio decreased from 7.6% to 4.5% with the combination method compare to blue dye method^15,19^. Moreover, inexperienced surgeons could achieve low false-negative rate with the combination method^15,19^.

The migration rate of blue dye and radiotracer through the lymphatic system is, however, inconsistent. Thus, the combination method fails to achieve complementarity between the visual assessment and radioactivity and requires separate injection of the blue dye and radiotracer. The radiotracer is injected before surgery and the blue dye, during surgery. Therefore, there is an unmet need for the development of a multifunctional agent with blue color and radioactivity that will accumulate into SLN.

**Figure 1:**
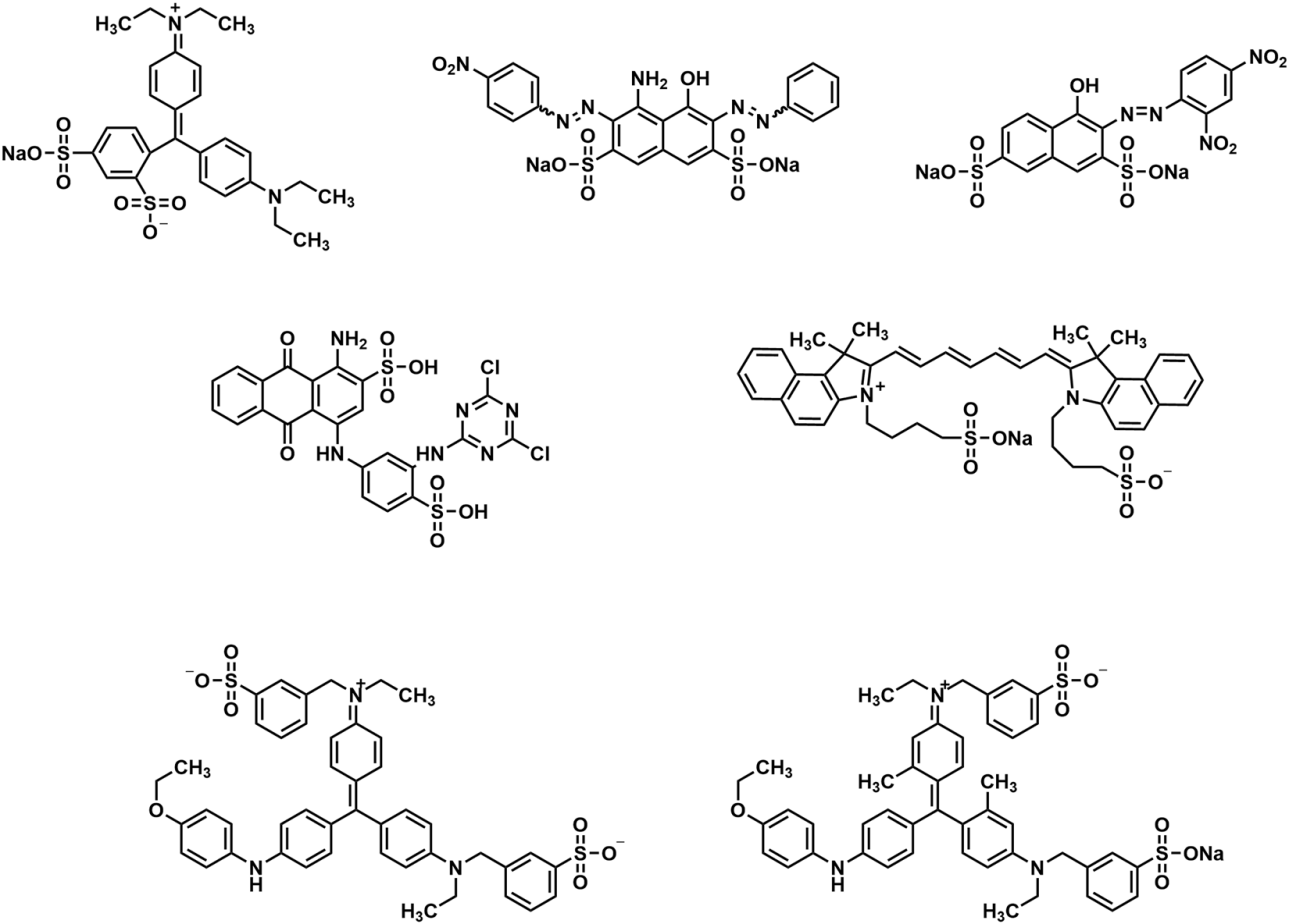
Molecular structure of dyes. Patent blue VF (PBVF), naphthol blue black (NBB), nitrazine yellow (NY), reactive blue 4 (RB4), indocyanine green (ICG), brilliant blue R (BR), brilliant blue G (BG).

In the present study, we aimed to develop radio-nanocarriers conjugated with blue dyes. Various blue dyes that bind to albumin and MSA with high affinity and exhibit a strong dark blue color, such as naphthol blue black (NBB), patent blue VF (PBVF), reactive blue 4 (RB4), nitrazine yellow (NY), indocyanine green (ICG), brilliant blue R (BR), and brilliant blue G (BG) (Fig. 1), were tested and NBB showing the highest binding was selected as the best candidate. We prepared ^99m^Tc-MSA-NBB conjugate for SLN detection *in vivo* by visual investigation, fluorescence imaging, and single photon emission computed tomography (SPECT)/computed tomography (CT).

## Results

### Confirmation of binding efficiencies

**Figure 2:**
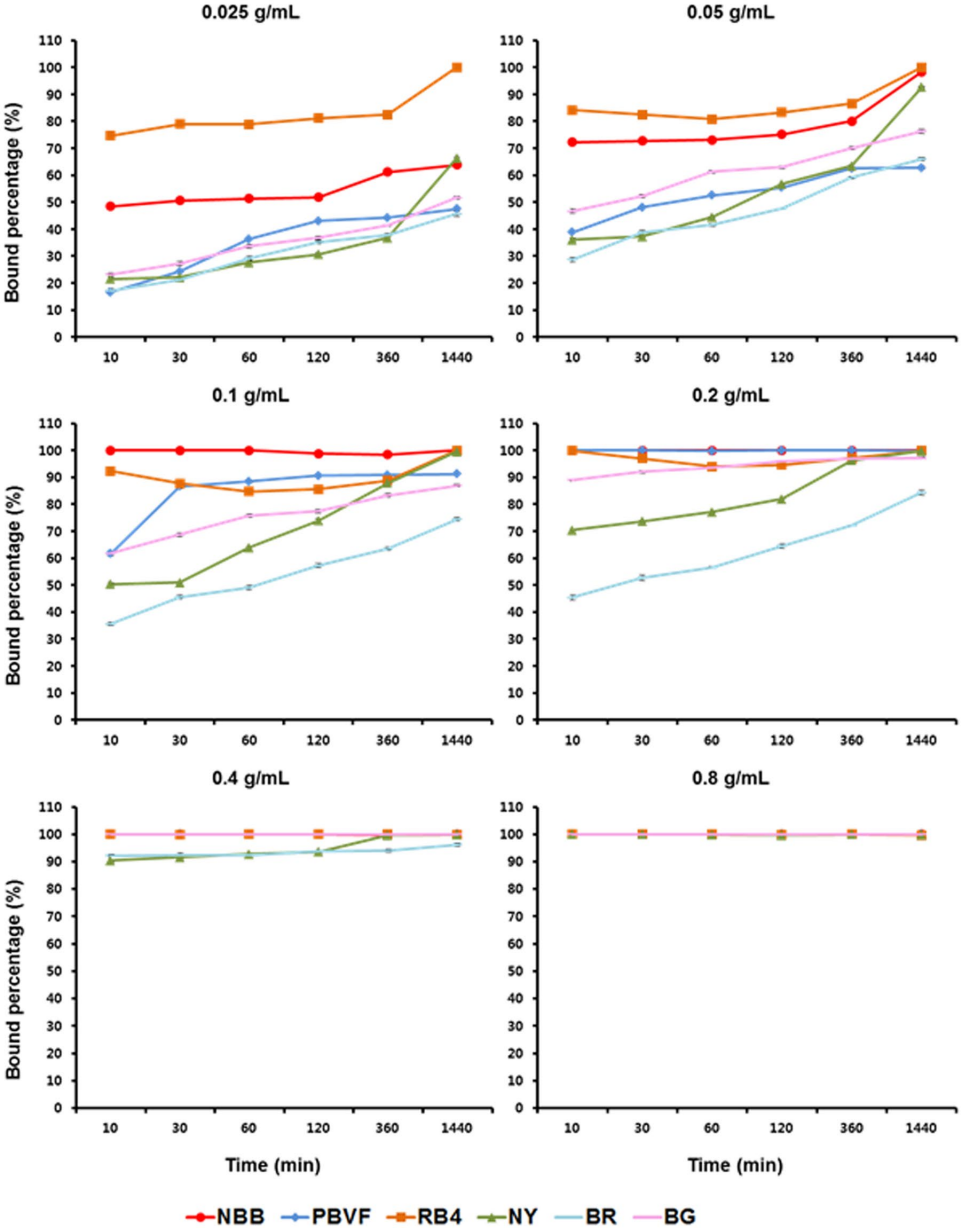
Binding of MSA and dyes determined by TLC (n = 3, mean ± SD). Each 0.33 mM dye was incubated with 0.025~0.8 g/mL MSA.

Binding efficiencies of MSA and various dyes were determined by TLC after reacting MSA with dyes at 37 °C. Binding efficiencies of all dyes increased with time until 24 h, which evidences the stability *in vitro* at least for 24 h (Fig. 2). ICG was invisible on TLC at 1 mM concentration, and no results were obtained. The required concentration of MSA for 100% binding of NBB, PBVF, RB4, BG, NY, and BR was 2.5, 5, 5, 10, 20, and 20 mg, respectively (Fig. 2). NBB required the least amount of MSA (2.5 mg) for 100% binding, which was the highest binding affinity among all the tested dyes. The MSA-NBB conjugate showed a clean single peak on HPLC representing a molecular weight between 44,000 and 158,000 Da (Supplementary Figure S2). The results of dynamic light scattering (DLS) analysis showed that the particle size of MSA (6.34 ± 0.49 nm) was larger than HSA (5.58 ± 0.16 nm) due to conjugated mannose groups, and it increased to 9.32 ± 0.34 nm after ^99m^Tc-labeling due to the reduction of disulfide bonds (Supplementary Figure S3). However, we couldn’t obtain the DLS data of dye-conjugates of ^99m^Tc-MSA due to the interference of blue color and fluorescence.

### Absorption spectra and molar absorption coefficient of MSA-dye conjugates

**Figure 3:**
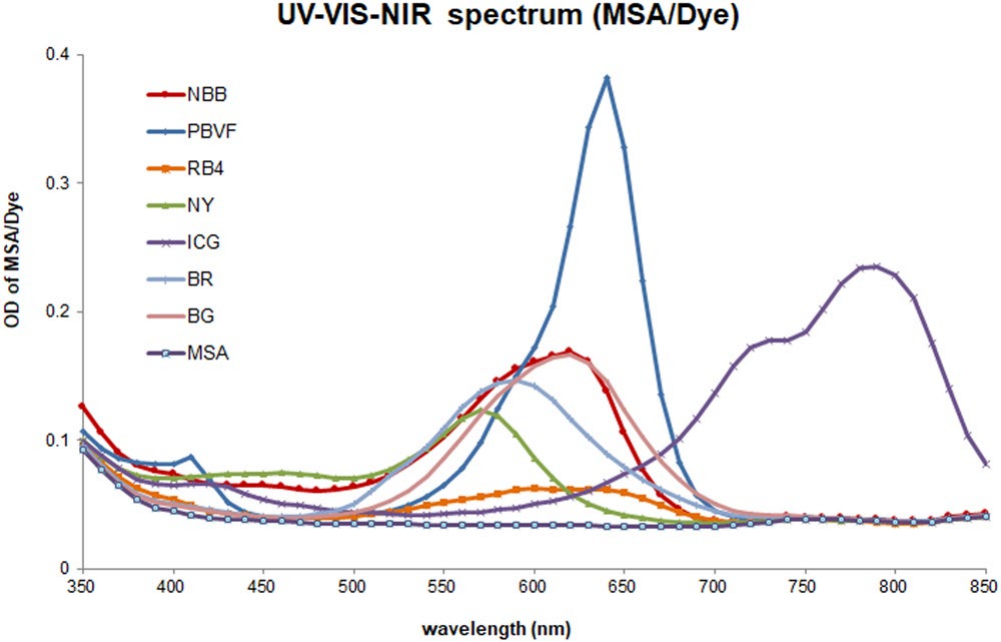
Spectra of MSA-dye conjugates scanned from 350 to 850 nm wavelengths.

We obtained UV-VIS-NIR spectra of MSA-dye conjugates from 350 to 850 nm wavelengths (Fig. 3). MSA itself showed no peak in the scanned range, while MSA-PBVF displayed a peak at 640 nm (Fig. 3). MSA-NBB showed a peak at 620 nm with an OD value of 0.168. Values of ε were calculated with Beer-Lambert law using OD values at peak wavelength, dye concentration (5 µM), and cell length (0.54 cm) (Table 1). MSA-PBVF demonstrated the highest ε value of 141,481 M^−1^·cm^−1^, while ε value of MSA-NBB was 62,222 M^−1^·cm^−1^ (Table 1).

**Table 1:** Optical Values of Dyes after Conjugation with MSA.

Dye (nm)	NBB	PBVF	RB4	NY	ICG	BR	BG
Peak (nm)	620	640	600	560	790	590	620
OD	0.168	0.382	0.063	0.117	0.235	0.147	0.167
ε (M^−1^·cm^−1^)	62222.2	141481	23333.3	43333.3	87037	54444.4	61851.9

### *In vitro* visibility test

**Figure 4:**
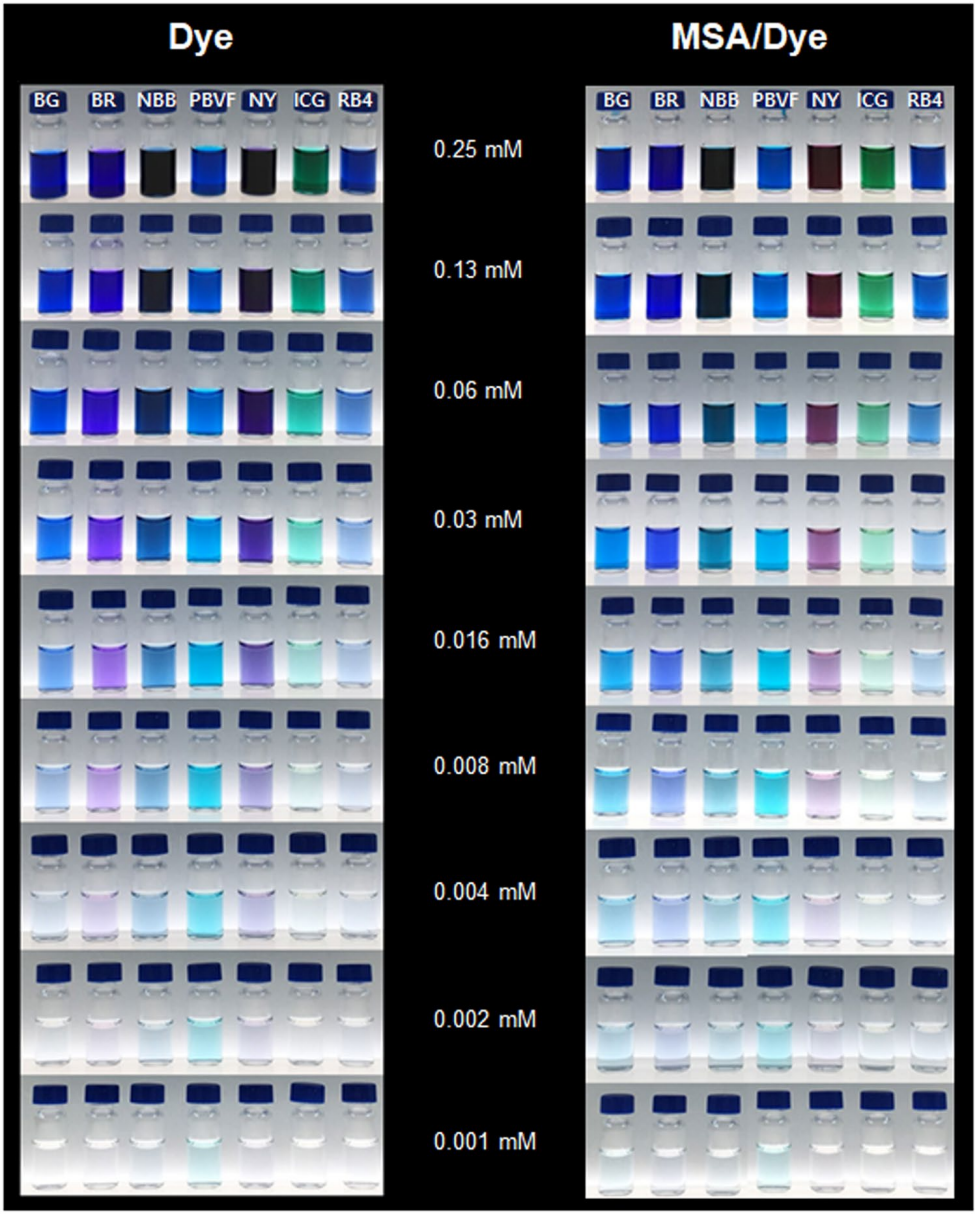
*In vitro* visibility test of dyes before and after conjugation with HSA. The samples were serially diluted from 0.25 to 0.001 mM.

Dyes and MSA-dye conjugates were serially diluted for visibility monitoring (Fig. 4). No clear distinguishable visibility was observed for the dyes and MSA conjugate at the same concentration (Fig. 4). NBB and NY showed the strongest color at high concentration (0.25 mM); however, PBVF was most visible at low concentration (0.001–0.004 mM). No strong correlation was observed between visibility and ε value.

### *In vivo* visible and fluorescence images

**Figure 5:**
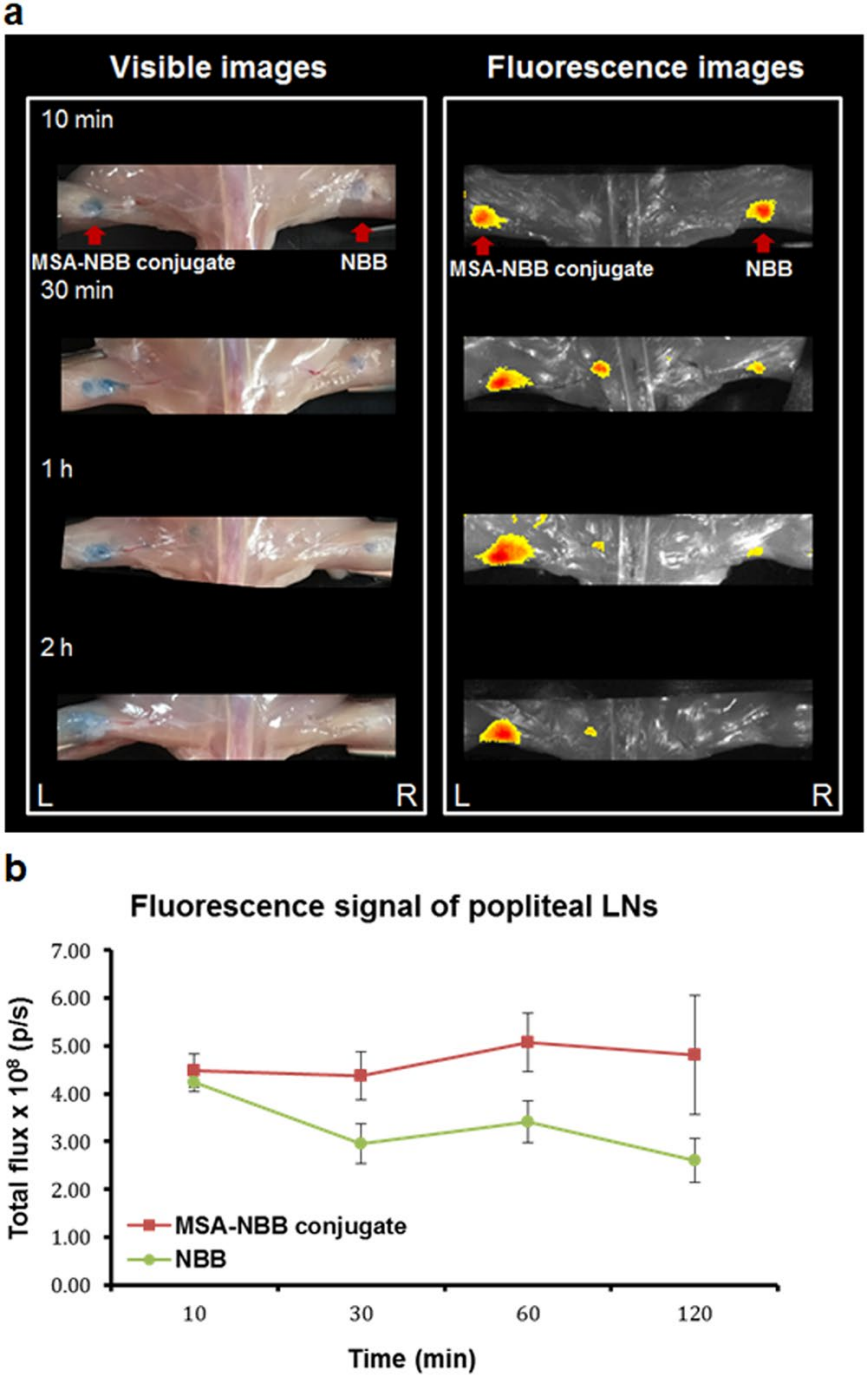
(**a**) Visible and fluorescence images of mice subcutaneously injected with ^99m^Tc-MSA-NBB and NBB at the left and right footpad, respectively (n = 3). The images were obtained at 10 min, 30 min, 1 h, and 2 h after injection. (**b**) Photon flux from fluorescence images were plotted by time. Vertical bars represent SD.

The conjugate ^99m^Tc-MSA-NBB was subcutaneously injected in the left footpads of mice and its accumulation in popliteal LN was observed at 10 min (Fig. 5a). Popliteal LN showed blue color after removing the skin around it. The resected popliteal LN showed fluorescence signal. The uptake was maintained at least for 2 h after injection. NBB injected in the right footpad also showed popliteal LN uptake at 10 min (Fig. 5a). However, the uptake was low and decreased rapidly with time.

Fluorescence images were obtained from the same mouse and showed consistency with visible observation (Fig. 5a). We quantified the fluorescence signal of the popliteal LN uptake. The photon flux of ^99m^Tc-MSA-NBB conjugate and NBB accumulated in the popliteal LN at 10 min were 4.11 × 10^8^ s^−1^ and 4.03 × 10^8^ p/s, respectively. While the fluorescence of ^99m^Tc-MSA-NBB conjugate at the popliteal LN was maintained for 2 h, the fluorescence of NBB rapidly decreased (Fig. 5b). ^99m^Tc-MSA-NBB conjugate demonstrated about two-fold higher popliteal LN uptake as compared with NBB from 30 min to 2 h after footpad injection.

### Analysis of SPECT/CT

**Figure 6:**
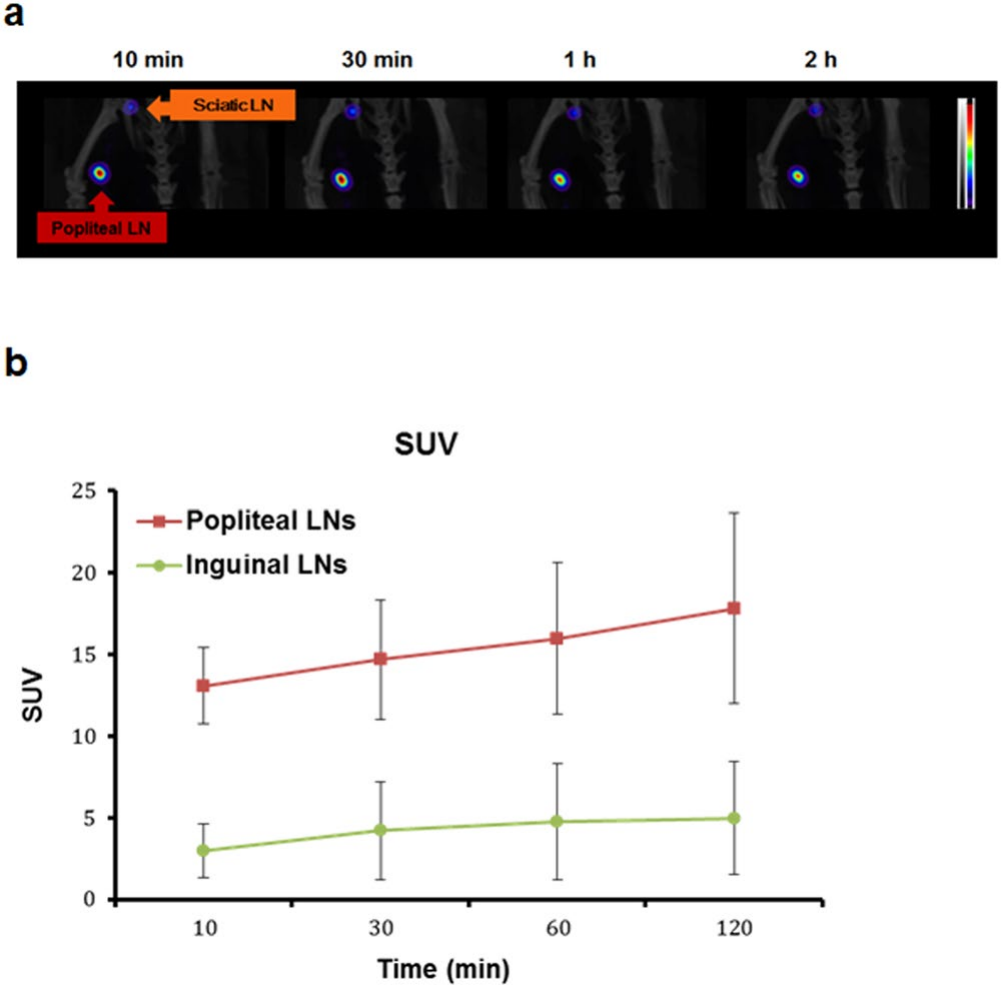
(**a**) SPECT/CT was obtained at 10 min, 30 min, 1 h, and 2 h after ^99m^Tc-MSA-NBB conjugate injection into the left footpad of mice (n = 3). (**b**) SUVs of popliteal LN by time was plotted. Vertical bars represent SD. SUVs are almost constant until 2 h of footpad injection. Popliteal LN uptake value of ^99m^Tc-MSA-NBB conjugate was about five-fold higher than inguinal LN uptake value at all time points.

We found that ^99m^Tc-MSA-NBB conjugate showed radiochemical purity of over 99%. SPECT/CT was obtained at 10 min, 30 min, 1 h, and 2 h after ^99m^Tc-MSA-NBB conjugate injection into the left footpads of mice. SPECT/CT images showed the uptake of ^99m^Tc-MSA-NBB at the popliteal and inguinal LN (SUV 10.72 and 1.99, respectively) at 10 min and the signal was maintained (SUV 11.56 and 2.0, respectively) until 2 h after injection (Fig. 6). SPECT/CT results revealed that the popliteal LN uptake of ^99m^Tc-MSA-NBB conjugate was about five-fold higher than inguinal LN uptake at all time points.

Popliteal LNs were removed after SPECT/CT studies. We confirmed the complete resection of popliteal LN by repeated SPECT/CT showing no remaining radioactivity around the resected area after removal of popliteal LN.

## Discussion

Here, we aimed to develop a multifunctional imaging agent and tested its feasibility for SLN mapping in normal mice. SLN mapping is important for image-guided surgery of breast cancer and melanoma.

^99m^Tc-MSA, developed for SLN imaging^10^, was shown to bind to the mannose receptor on the macrophage surface^20–22^. Thus, ^99m^Tc-MSA may serve as an excellent agent for SLN imaging. Although radionuclide imaging provides important information on the position of SLN, the dissection technique depends on the operator’s intuition because radioactivity is detected only with gamma probes during surgery.

Various blue dyes are known to bind strongly to albumin and accumulate to SLN and thus be helpful to locate SLN during dissection. However, SLNs are deep inside the tissues and, hence, invisible before dissection. Moreover, blue dyes easily diffuse and pose difficulty in SLN detection. In this context, the multimodal ^99m^Tc-MSA-dye conjugate could be an excellent solution.

To prepare the multimodal agent, we tested the binding ability of various dyes such as NBB, PBVF, NY, RB4, ICG, BG, and BR to MSA. Among these, NBB showed the highest binding ability to MSA (Fig. 2). Although the molar absorption coefficient was not the highest, visibility test showed excellent results. In spite of the fact that the molar absorption coefficient and visibility failed to show a strong correlation, the visibility is more important in practical applications. Thus, NBB was selected for the animal experiment.

The current two-step preparation procedure might be more simplified by producing kits to prepare ^99m^Tc-MSA-NBB by simple one-step addition of ^99m^Tc. It might require systematic study to adjust the amount of MSA and NBB.

Popliteal LN is the first LN observed after the dye injection into the mouse footpad and may be considered as an SLN. In preliminary studies, NBB was shown to emit fluorescence. Thus, the fluorescence study was performed together with the visible study in animal experiment. Results of the animal study confirmed that popliteal LN was easily identified by MSA-NBB conjugate, which was sustained for at least for 2 h. Furthermore, fluorescence studies revealed strong fluorescence signal from MSA-NBB conjugate, which accumulated in the popliteal LN from 10 min to 2 h after footpad injection. From visible and fluorescence studies, we confirmed that MSA-NBB conjugate was targeted to the popliteal LN. It is possible to prevent dye diffusion and increase its accumulation in the LN by conjugating it with MSA. However, LNs under the skin or tissue are invisible both in visibility and fluorescence studies. The use of radioisotope ^99m^Tc can overcome this issue due to its highly penetrating gamma-ray.

The use of dye emitting near-infrared (NIR) fluorescence may allow visualization of LNs that are located deeper inside the tissue. ICG has been used for the detection of SLN by NIR imaging^23,24^. ICG-MSA conjugate is known to prevent the diffusion of ICG and has shown improved uptake into SLN^25,26^. However, NIR imaging requires a special instrument to observe fluorescence. Thus, NIR images are observed always using a monitor, while blue dyes can be visualized directly with naked eyes, thereby facilitating the dissection of LN.

Results of SPECT/CT were in line with those of visible and fluorescence results. ^99m^Tc-MSA-NBB conjugate was taken up into popliteal LN specifically at 10 min and maintained for 2 h after footpad injection. Moreover, we demonstrated inguinal LN uptake, which was absent in visual and fluorescence imaging. Thus, ^99m^Tc-MSA-NBB conjugate accumulated specifically in SLN and showed very low uptake in the next LN. SPECT/CT was excellent in quantification and its uptake in popliteal LN was easily estimated to be about five-fold higher than that in inguinal LN at all time points.

When we removed the skin of the mouse leg, we could easily identify popliteal LN owing to the blue color of MSA-NBB, and the popliteal LN was easily dissected. SPECT/CT was helpful to identify the positioning of LN before surgery and quantification. Fluorescence was more sensitive than visual monitoring.

According to the above results, the sensitivity of the modalities could be in the order of SPECT/CT, fluorescence, and visible monitoring. Although the sensitivity of visible monitoring is low, it provides convenience and accuracy to dissect SLN.

Image-guided surgery is gathering more attention and the development of multimodal SLN-detecting agents will provide more advanced options for future progress in surgery. For instance, a multimodal imaging agent for SLN detection based on nanoparticles was developed for the simultaneous imaging with positron emission tomography (PET) and magnetic resonance imaging (MRI)^27^.

In this study, we developed a multimodal imaging agent, ^99m^Tc-MSA-NBB conjugate, for SLN mapping that may be assessed by visual monitoring, fluorescence imaging, and SPECT/CT. We confirmed that ^99m^Tc-MSA-NBB conjugate bound specifically to, and accumulated in, popliteal LN after footpad injection. We found that the visual assessment was convenient and fluorescence imaging demonstrated higher sensitivity. SPECT/CT allowed more accurate quantification and positioning before surgery. Thus, ^99m^Tc-MSA-NBB conjugate has a great potential for SLN mapping under clinical settings.

## Materials and Methods

Human serum albumin (HSA) solution (20%) was obtained from Green Cross Corporation (Seoul, Korea). PBVF, NY, RB4, BR, BG, and α-d-mannopyranosylphenyl isothiocyanate were purchased from Sigma-Aldrich (St. Louis, MO, U.S.A.) and NBB and ICG, from Tokyo chemical industry Co. (TCI, Japan). All other reagents and solvents were supplied by Sigma-Aldrich (St. Louis, MO, U.S.A.).

Sephadex G-25 columns (PD-10, Pharmacia, Uppsala, Sweden) were used for the purification of MSA. Thin-layer chromatography (TLC) silica gel 60F_264_ (Sigma-Aldrich, St. Louis, MO, U.S.A.) was used for the determination of binding efficiencies. Instant thin-layer chromatography silica gel (ITLC-SG) plates were procured from Agilent Technologies (Santa Clara, CA, U.S.A.).

Fujifilm LAS-3000 was purchased from Fujifilm Life Science (West Avenue, Stamford, U.S.A.). Varioskan Flash (Thermo Fisher scientific Inc., Waltham, Massachusetts, U.S.A.) was used for UV-VIS-NIR screening. Fluorescence images were obtained with IVIS Lumina II (Caliper Life Science, Hopkinton, Massachusetts, U.S.A.) and Igor Pro 4.09 A (Wave Metrics Inc., Portland, Oregon, U.S.A.). ^99m^Tc-pertechnetate was eluted from ^99^Mo/^99m^Tc-generator (Sam Young Unitech Co., Korea). Bio-Scan AR-2000 scanner (Bioscan, WI, U.S.A.) was used for the measurement of radiochemical purity. SPECT/CT were obtained using NanoSPECT/CT^Plus^ (Mediso, Budapest, Hungary) and analyzed with DICOM browser in InVivoScope (IVS) program.

Animals. Male BALB/c mice (4-week-old) were purchased from OrientBio (Seoul, Korea) and mice were housed in at the Seoul National University Hospital (Seoul, Korea), which was accredited by AAALAC International (2007, Association for Assessment and Accreditation of Laboratory Animal Care International). Mice were housed in 5 animals per cage at 22 ± 2 °C, humidity of 40–60% and 12 h light/dark cycle. All of the animal studies were approved by Institutional Animal Care and Use Committee of the Clinical Research Institute and performed in accordance with the National Research council guidelines from the institute.

### Preparation of MSA and kits for ^99m^Tc labeling

We prepared MSA as previously described with minor modification^28^. Briefly, HSA (20 mg) and α-d-mannopyranosylphenyl isothiocyanate (5.5 mg) were added to 5 mL of 0.1 M sodium carbonate buffer (pH 9.5). The mixture was reacted at room temperature for 20 h with continuous stirring, followed by purification with PD-10 size-exclusion column using distilled water (DW). The purified MSA was freeze-dried and stored at −20 °C until analysis.

We prepared MSA kit for ^99m^Tc labeling according to the previously described procedure with minor modification^10^. Briefly, HSA (10.7 mg) was dissolved in 1 mL of 0.1 M sodium carbonate buffer (pH 9.5) and treated with α-d-mannopyranosylphenyl isothiocyanate (1 mg) at room temperature for 20 h with continuous stirring. For reduction of MSA, 40 μL of 0.3 M ethylenediaminetetraacetic acid (EDTA, pH 8.0), 40 μL of 1 M sodium bicarbonate, and 50 μL of 1.5 M β-mercaptoethanol was added. The reaction mixture was incubated at 37 °C for 1 h and purified with PD-10 column using phosphate buffer (pH 6.0). The solution was aliquoted into vials containing 1 mg MSA, 0.25 mg sodium medronate, 80 μg sodium ρ-aminobenzoate, and 13.6 μg stannous fluoride per vial. The vials were freeze-dried and store at −20 °C for further study.

### Calculation of binding efficiencies *in vitro*

Binding efficiencies of MSA and various dyes were tested by treating 25 μL of 1 mM dye solution in DW with 0.625, 1.25, 2.5, 5, 10, and 20 mg MSA solution in 50 μL of DW. The mixture was incubated at 37 °C under continuous stirring and 0.3 μL aliquot of each mixture was spotted on TLC plate at 10 min, 30 min, 1 h, 2 h, 6 h, and 24 h after incubation. Vacuum chamber was used for drying TLC plate over 40 min before TLC development. Methanol (MeOH) and dichloromethane (DCM) was used as a TLC eluent at 3:7 ratio for PBVF, NBB, ICG, and BR and 1:4, 1:3, and 8:5 ratio for BG, NY, and RB4, respectively. MSA-bound dyes remained at the origin and free dyes moved with the solvent to front line. TLC plates were scanned by Fujifilm LAS 3000 and quantified by multi-gauge 3.0. Binding efficiencies were calculated from the obtained data.

### Determination of the molar absorption coefficient (ε)

We conducted UV-VIS-NIR spectrum assay to determine ε values. Each 25 µL of 1 mM dye solution in DW was added to 5 mg MSA solution in 50 µL of DW. The mixture was incubated at 37 °C for 24 h under continuous stirring. The optical density (OD) was measured by Varioskan Flash screening mode at 350–850 nm. Values of ε were calculated according to Beer-Lambert Law. Light path was 0.54 cm and concentration of various dyes was 5 µM.

$$\begin{array}{c}{\rm{\varepsilon }}={\rm{A}}/{\rm{L}}/{\rm{C}}\\ {\rm{A}}={\rm{optical}}\,{\rm{density}}\\ {\rm{L}}={\rm{light}}\,\mathrm{path}-\mathrm{length}\,{\rm{of}}\,{\rm{solution}}\,({\rm{cm}})\\ {\rm{C}}={\rm{concentration}}\,{\rm{of}}\,{\rm{solution}}\,({\rm{M}}){\rm{.}}\end{array}$$

### *In vitro* visibility test of dyes

Visible monitoring was conducted to investigate the dye that displays the darkest blue color after binding with albumin. Each

mL of 1 mM dye solution in DW and 2 mL of 20% MSA in DW was added to 1 mL of DW. The mixture was incubated at 37 °C for 2 h with continuous stirring. The reaction mixture was serially diluted with DW from 0.25 to 0.0001 mM. Each dye solution without MSA was prepared for comparison. The prepared solutions were monitored by visual inspection.

### Preparation of ^99m^Tc-MSA-NBB conjugate

Two milliliters containing 25.6 MBq of ^99m^Tc-pertechnetate was added to the above prepared MSA kit and reacted at room temperature for 30 min. Radiochemical purities were checked using ITLC-SG/Umezawa (ethanol:10% ammonium acetate = 1:1). ^99m^Tc-MSA remained at the origin and unlabeled ^99m^Tc moved with the solvent to the front (Supplementary Figure S1A). The formation of reduced hydrolyzed ^99m^Tc remaining at the origin was checked by paper chromatography impregnated with 5% bovine serum albumin (BSA)/normal saline (Supplementary Figure S1B). The radioactivity on ITLC-SG plate was scanned and quantified using Bio-Scan AR-2000 scanner. The labeling efficiency was over 99%. To make MSA-NBB conjugate, MSA (25 mg) in 50 μL of DW was added to 25 μL of 10 mM NBB solution. The mixture was incubated at 37 °C for 2 h with continuous stirring. To prepare ^99m^Tc-MSA-NBB conjugate, 30 μL of MSA-NBB conjugate was mixed with 5 μL of ^99m^Tc-MSA (25.6 MBq).

### Animal experiment for the detection of LNs by visible, fluorescence, and radioactivity analyses

Animal studies were performed according to the National Research council guidelines from the Seoul National University Hospital. The feasibility of ^99m^Tc-MSA-NBB conjugate for visible and fluorescence imaging of LNs was tested with male BALB/c mice (5-week old, n = 3). Briefly, 30 μL of the prepared MSA-NBB conjugate was subcutaneously injected into the left footpad of the mouse anesthetized with 2% (v/v) isoflurane at 1 L/min oxygen flow. For comparison, 30 μL of NBB solution (3.33 mM NBB in DW) was injected into the right footpad of the same mouse. Visible and fluorescence images were obtained at 10 min, 30 min, 1 h, and 2 h after injection. Fluorescence images were obtained using IVIS Lumina II equipment (excitation/emission: 600/670 nm) with an exposure time of 1 s. The obtained fluorescence images were analyzed by LIVINGIMAGE version 2.12 (Xenogen) and IGOR version 1.24 (WaveMetrics) image analysis software.

Images for SPECT/CT were obtained at 10 min, 30 min, 1 h, and 2 h after injection using NanoSPECT/CT^Plus^ and the acquisition time was 20 min. The obtained SPECT/CT was analyzed with DICOM browser in InVivoScope (IVS) program. In order to calculate Standard Uptake Values (SUV) of LN, 3-dimensional regions of interest (3D ROI) were drawn on popliteal and inguinal LNs. SUVs were calculated by SUV calculator of the software using 3D ROI volume, 3D ROI radioactivity, mouse body weight, and injected dose.

$$\begin{array}{c}{\rm{SUV}}={\rm{AW}}/{\rm{V}}/{\rm{D}}\\ {\rm{A}}={\rm{3D}}\,{\rm{ROI}}\,{\rm{radioactivity}}\,({\rm{MBq}})\\ {\rm{W}}={\rm{mouse}}\,{\rm{body}}\,{\rm{weight}}\,({\rm{g}})\\ {\rm{V}}={\rm{3D}}\,{\rm{ROI}}\,{\rm{volume}}\,({\rm{mL}})\\ {\rm{D}}={\rm{injected}}\,{\rm{dose}}\,({\rm{MBq}}).\end{array}$$

## Electronic supplementary material

Supplementary material
