# Supplementary material for: Naphthol Blue Black and 99mTc-Labeled Mannosylated Human Serum Albumin (99mTc-MSA) Conjugate as a Multimodal Lymph Node Mapping Nanocarrier
Source: Sci Rep. 2018 Sep 11;8:13636. doi: 10.1038/s41598-018-31933-1 (PMC6134003; doi:10.1038/s41598-018-31933-1)
Supplement: Supplementary file 1 — Supplementary material [file 41598_2018_31933_MOESM1_ESM.pdf]

Supplementary material

Naphthol Blue Black and ^{99m}Tc-Labeled Mannosylated Human Serum Albumin (^{99m}Tc-MSA) Conjugate as a Multimodal Lymph Node Mapping Nanocarrier

Ji Youn Lee[1, 2], Ho Young Kim[1], Yun-Sang Lee[1], and Jae Min Jeong[1, 2, 3]

Affiliation

[1]Department of Nuclear Medicine, Institute of Radiation Medicine, Seoul National University College of Medicine, Seoul, Republic of Korea

[2]Department of Biomedical Sciences, Seoul National University Graduate School, Seoul, Republic of Korea

[3]Cancer Research Institute, Seoul National University, Seoul, Republic of Korea

Contents

Supplementary Figure S1. Chromatography of ^{99m}Tc-MSA to check the radiochemical purity.

Supplementary Figure S2. Characterization of NBB and MSA conjugate by size-exclusion HPLC.

Supplementary Figure S3. Particle size distribution of HSA, MSA and ^{99m}Tc-MSA analyzed by DLS.

Supplementary Figure S4. Visible image, SPECT/CT and optical image were obtained using same mouse 4 h after footpad injection to confirm the stability of ^{99m}Tc-MSA-NBB conjugate *in vivo*.

Supplementary figure 1

a

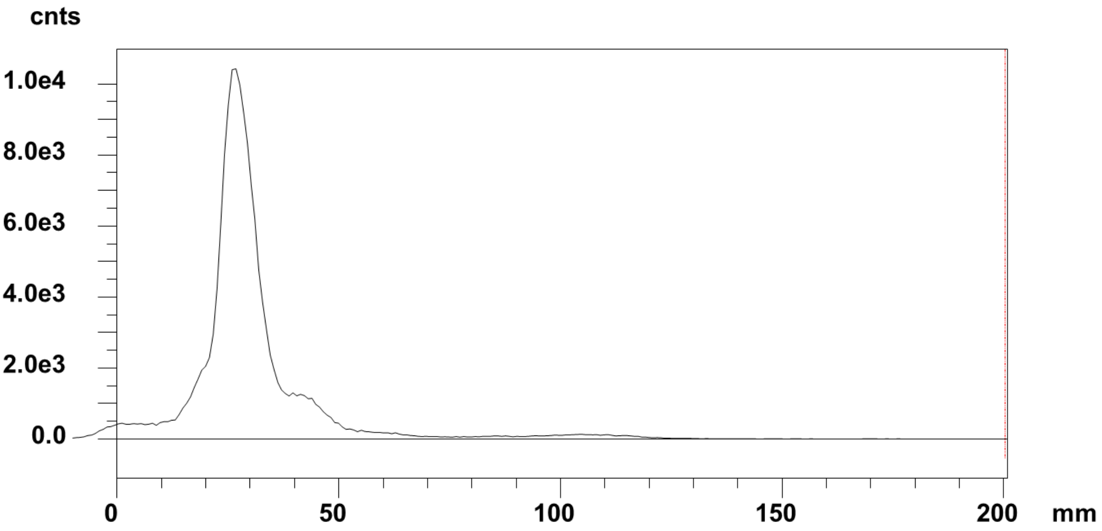

b

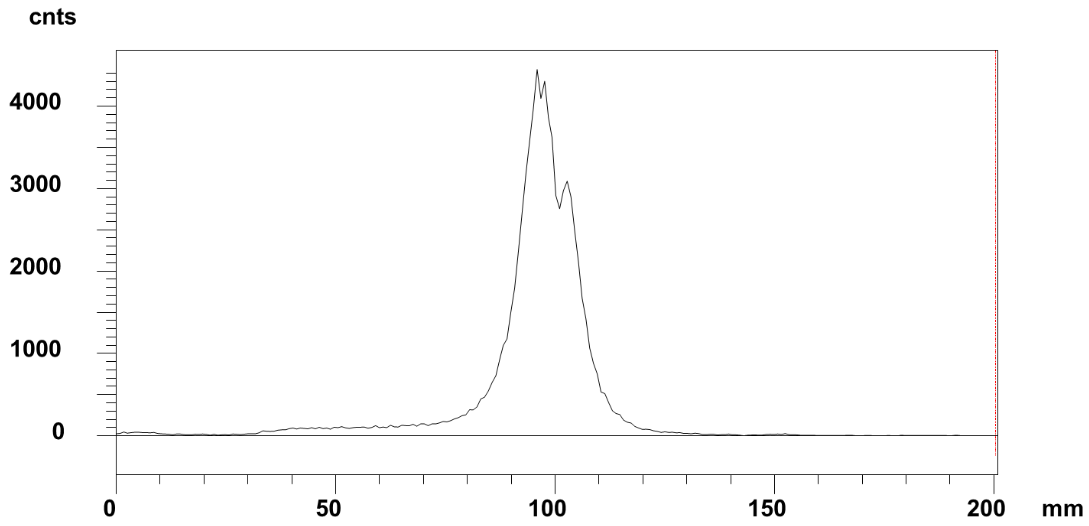

Supplementary Figure S1. Chromatography of ^{99m}Tc-MSA to check the radiochemical purity. (A) ^{99m}Tc-MSA remained at the origin and unlabeled ^{99m}Tc moved with the solvent to the front (ITLC-SG/Umezawa (ethanol:10% ammonium acetate = 1:1)). (B) All the radioactivity moved to the front except [^{99m}Tc]TcO_2 (paper chromatography impregnated with 5% BSA/normal saline). ^{99m}Tc-MSA-NBB conjugate showed radiochemical purity of over 99%.

Supplementary figure 2

a

b

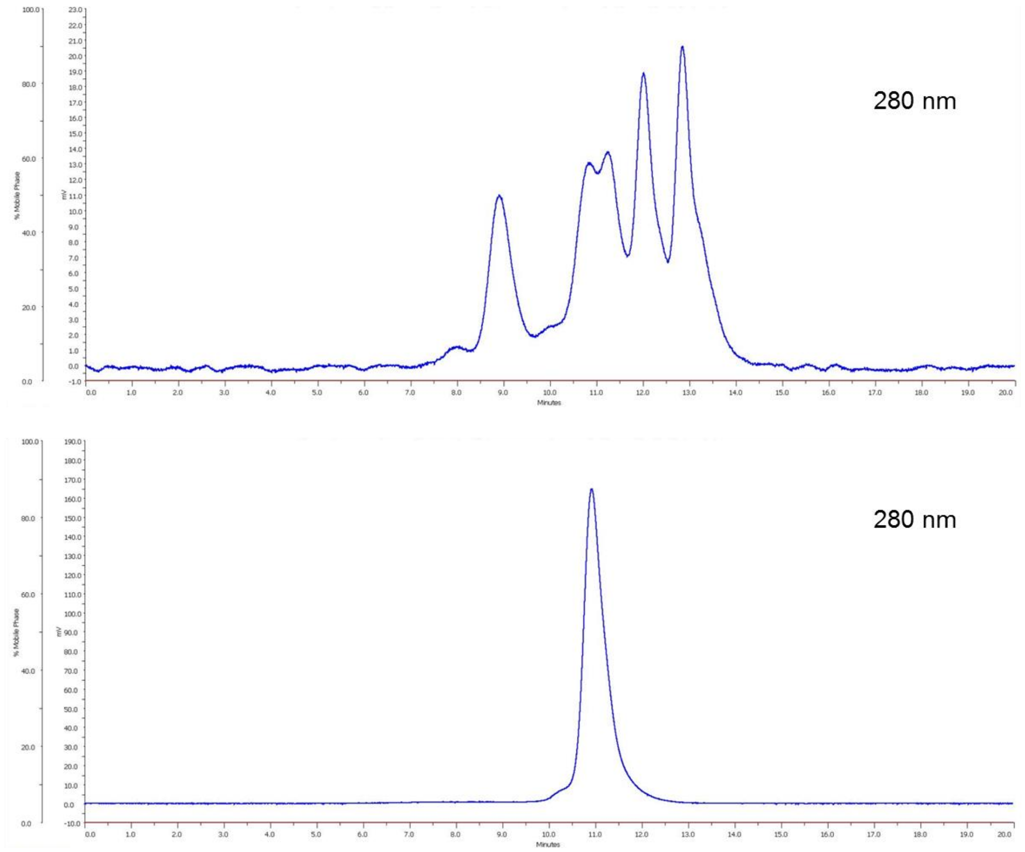

Supplementary Figure S2. Characterization of NBB and MSA conjugate by size-exclusion HPLC. (A) Gel filtration standard (BIO-RAD) was used for standard at 280 nm; aggregated protein 5.0-7.0 min, thyroglobulin (MW 670,000) 8.91 min, γ-globulin (MW 158,000) 10.72 min, ovalbumin (MW 44,000) 11.3 min, myoglobin (MW 17,000) 12.02 min, vitamin B12 (MW 1,350) 12.86 min. (B) The retention time of MSA-NBB conjugate was 10.92 min at 280 nm, expectably. (TSKgel G4000SW$_{XL}$, 7.8 x 300 mm, 100% 0.1 M sodium phosphate buffer pH 6.7, isocratic from 0 to 20 min, flow rate 1 mL/min)

Supplementary figure 3

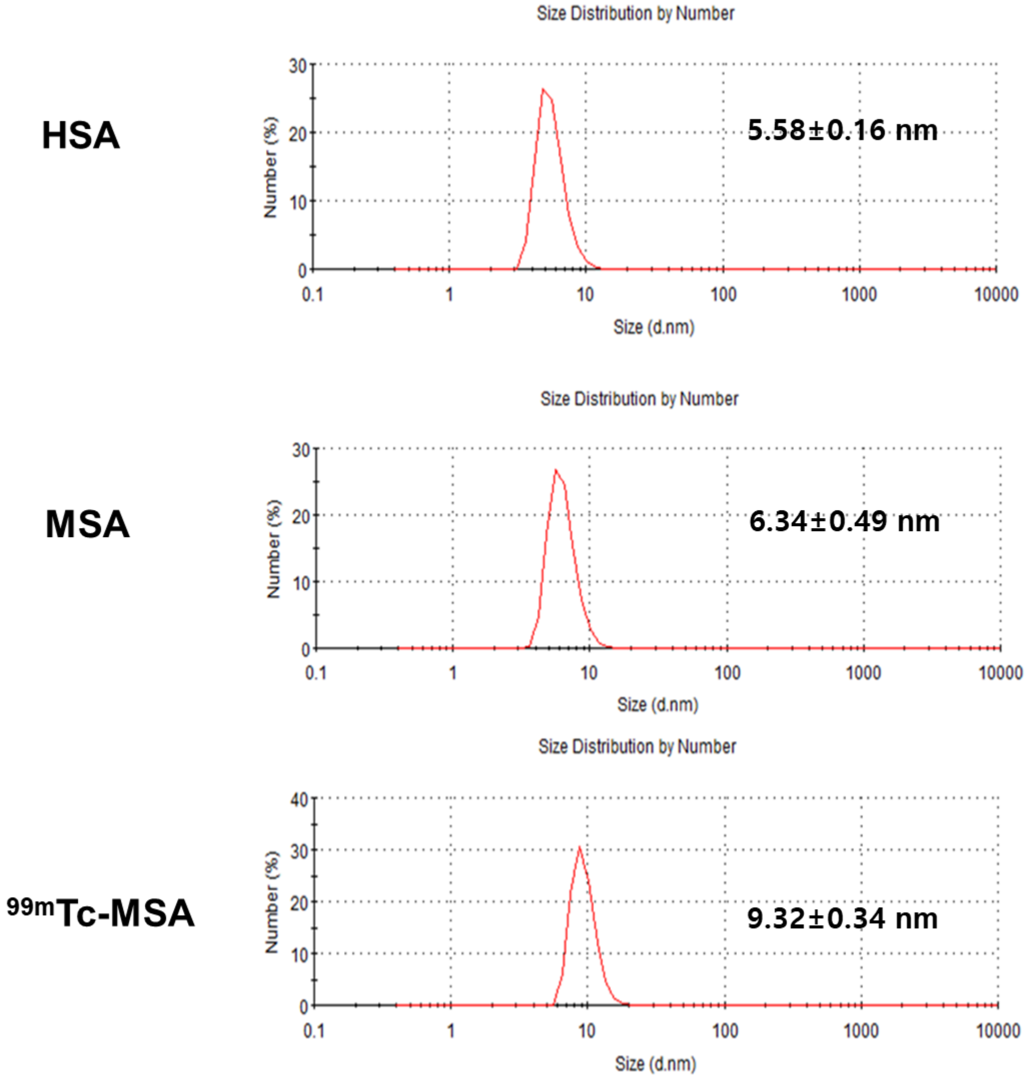

Supplementary Figure S3. Particle size distribution of HSA, MSA and ^{99m}Tc-MSA analyzed by DLS. Particle size of HSA was 5.58 nm which increased to 6.34 nm after conjugation with mannoses. The particle size of MSA increased to 9.32 nm after labeling with ^{99m}Tc due to the reduction of disulfide bonds. DLS analysis of NBB conjugated ^{99m}Tc-MSA was not possible because of the interference of blue color and fluorescence.

Supplementary figure 4

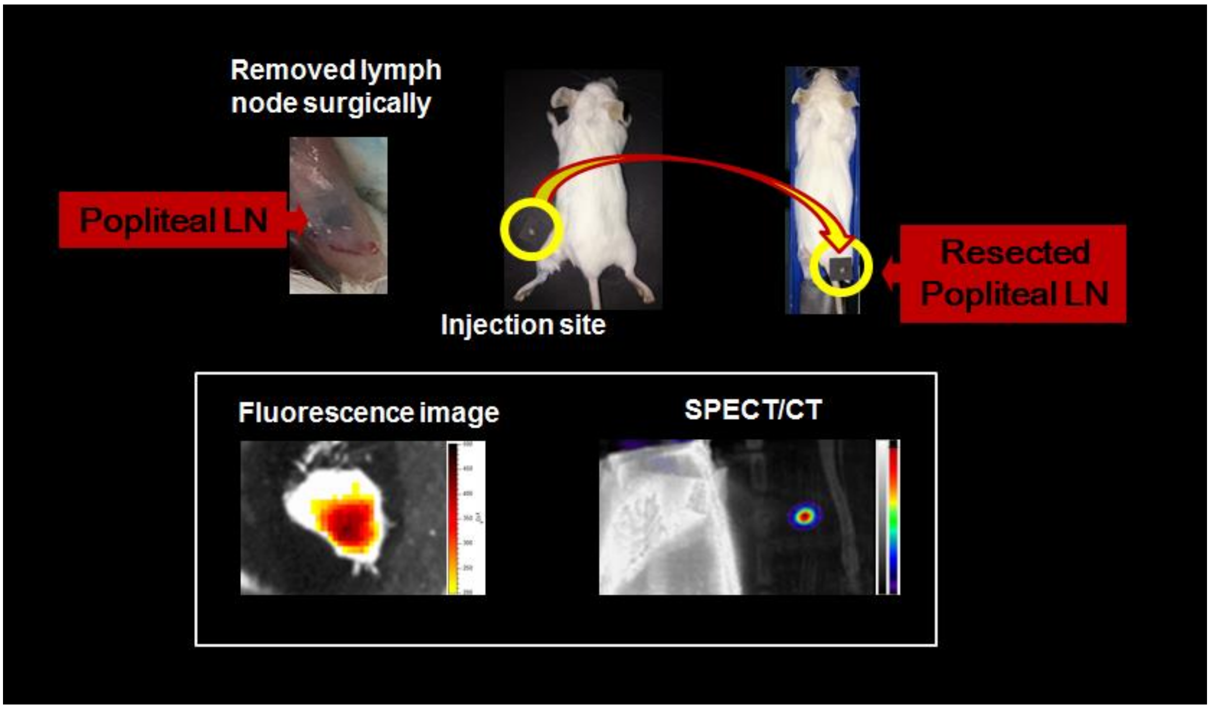

Supplementary Figure S4. Visible image, SPECT/CT and optical image were obtained using same mouse 4 h after footpad injection to confirm the stability of ^{99m}Tc-MSA-NBB conjugate *in vivo*. Skin which was near to popliteal LN was resected, and we were able to visualize the stained LN by MSA-NBB conjugate 4 h after footpad injection. The popliteal LN was resected solely based on the blue color and placed a resected LN on the opposite leg of the injection site. After then, SPECT / CT and optical images were obtained. We were able to confirm that the popliteal LN was clearly resected by SPECT/CT, and fluorescent signal was also observed in the removed LN.
